# Available evidence suggests that prevalence and risk of female genital cutting/mutilation in the UK is much lower than widely presumed - policies based on exaggerated estimates are harmful to girls and women from affected communities

**DOI:** 10.1038/s41443-021-00526-4

**Published:** 2022-01-15

**Authors:** Saffron Karlsen, Janet Howard, Natasha Carver, Magda Mogilnicka, Christina Pantazis

**Affiliations:** 1 Associate Professor in Sociology, Centre for the Study of Ethnicity and Citizenship, School of Sociology, Politics and International Studies, University of Bristol, Postal address: 11 Priory Road, Bristol, BS8 1TU UK; 2 Research Associate, Department of Anthropology, University of Bristol, Bristol, UK; 3 Lecturer in International Criminology, School for Policy Studies, University of Bristol, Bristol, UK; 4 Lecturer in Sociology, School of Sociology, Politics and International Studies, University of Bristol, Bristol, UK; 5 Professor of Zemiology, Centre for the Study of Poverty and Social Justice, School for Policy Studies, University of Bristol, Bristol, UK

**Keywords:** Quality of life, Health care

## Abstract

It is widely reported that ‘tens of thousands of girls’ are living in the UK with the risk of experiencing Female Genital Cutting or Mutilation (FGC/M). This paper reviews the data on which such claims are based. It finds that the data available with which to establish the scale of such risk is both sparse and problematic, and that the numbers claimed to be at risk are considerably over-inflated. For example, data collected by the National Health Service suggests that as few as eight girls had FGC/M while resident in the UK since their records began, with as few as one or two experiencing FGC/M types 1, 2 or 3. Other data publicly available or retrieved from Freedom of Information requests to the Home Office, Crown Prosecution Service, Ministry of Justice, Department for Education, National Health Service and academic sources also suggest that the ‘tens of thousands of girls’ claim is misplaced. Current UK FGM-safeguarding approaches, though well-intentioned, appear to be based on inaccurate estimates of FGC/M prevalence and risk. Existing research shows that these approaches directly harm communities, contributing to institutional discrimination, racially/religiously-motivated victimisation and the criminalisation of innocent families. This is an issue which must be urgently addressed.

Politicians and media sources claim ‘tens of thousands of girls’ in the UK are at risk of female genital cutting or mutilation (FGC/M; see Box 1 for definitions and terminology), despite growing academic evidence that the practice in the UK may be in decline [1–4]. Existing data available from the Home Office, Crown Prosecution Service, Ministry of Justice, Department for Education, National Health Service, and academic sources, reported here, similarly suggest that these claims are substantially overinflated. Moreover, the value of the data that are available is undermined by significant methodological problems. Policy must be based on accurate evidence. However, current FGM-safeguarding approaches, though well-intentioned, appear to be based upon inaccurate estimates of FGC/M prevalence and risk. These approaches directly harm communities, contributing to institutional discrimination, racially/religiously-motivated victimisation and the criminalisation of innocent families [5–7]. This is an issue which must be urgently addressed.

## Box 1

Definitions

According to the World Health Organisation (https://www.who.int/news-room/fact-sheets/detail/female-genital-mutilation), Female Genital Cutting/Mutilation (FGC/M) includes ‘all procedures that involve partial or total removal of the external female genitalia, or other injury to the female genital organs for non-medical reasons’. This definition includes several procedures including: clitoridectomy—the partial removal of the clitoris or prepuce (type 1); excision—the partial removal of the clitoris and labia minora (type 2); infibulation—the narrowing of the vaginal opening (type 3) and; any female genital piercing, pricking, incising, cauterising or scraping for non-medical reasons (type 4). FGM mandatory reporting duty in the UK includes female genital piercing (even when consented to by the person affected), tattooing and other procedures which are medically unnecessary.

Terminology

The term ‘female genital mutilation’ (FGM) is frequently used in policy and practice arenas. It is commonly used in relation to statutory processes relating to ‘FGM-safeguarding’, and as such we continue to use this phrase in this specific context. However, the phrase is controversial [8]. First, it is applied to a wide range of procedures, some of which are not associated with long-term tissue damage. Second, it typically excludes other procedures which are associated with long-term genital tissue damage, such as forms of cosmetic surgery and male circumcision. However, academic opinion is more diverse with some scholars preferring the term, female genital cutting (FGC) [8].

No national survey has been conducted with which to establish FGC/M prevalence in the UK. Instead, government policy, including FGM-safeguarding approaches, rely on estimates from a selection of relatively high FGC/M-prevalence countries from which people migrate to the UK. Such estimates suggest, for example, that 99% of women and girls living in Somalia have experienced FGC/M (https://data.unicef.org/resources/data_explorer/unicef_f/?ag=UNICEF&df=GLOBAL_DATAFLOW&ver=1.0&dq=SOM.PT_F_15-49_FGM.&startPeriod=1970&endPeriod=2021). However, the reliability of these data for establishing prevalence estimates even in those countries where the data are collected has been questionned [9]. This approach also ignores increasing evidence [10–12] that the likelihood of undergoing FGC/M is lower among those who are born in—or who have moved to—low FGC/M prevalence countries, compared with those living in high prevalence ones. This is particularly the case when an individual has moved at a young age to a low-prevalence country, even where their mother has already undergone FGC/M. The extrapolation of prevalence estimates and risk from one situation to another, without considering these processes of cultural change and how they may affect rates in post-migration locations may lead to a significant over-estimation of the level of risk in the UK context.

In the UK, several governmental departments collect information which could inform estimates of FGC/M prevalence and risk. Here we provide a summary of evidence from published or publicly-accessible sources or provided in response to requests for information under the Freedom of Information Act 2000 (FOIA) made by one of the authors to the Home Office (FOI 53514), Department for Education (FOI-2019-0016868) and Ministry of Justice (190624008) in April 2019.

One source of evidence concerns reports of FGC/M to the police. Research has established that there are barriers to reporting FGC/M to the police by women living in the UK who have been cut (including the belief that it would serve no purpose because they had experienced FGC/M as a child, and in other countries, as well their lack of trust in the police) [13]. Notwithstanding concerns that the introduction of new criminal laws has driven the practice underground making the FGC/M more difficult to detect [14], mandatory reporting requirements placed on healthcare professionals, social workers, and others by the Serious Crime Act 2015 should have counteracted some of this under-reporting. Yet despite this, the police in England and Wales (excluding Manchester Metropolitan police) recorded a total of 74 FGC/M offences in 2019–20, thirty-four of which were reported by professionals [15]. From this data source it is unlikely all instances of FGC/M in 2019–20 have been captured or that the FGC/M offences that have been recorded are indeed crimes in accordance with the law. Nevertheless, the mandatory recording of FGC/M is far below what would be expected given the above-cited claims of risk to ‘tens of thousands’ of girls.

Home Office evidence provided through Freedom of Information Act 2000 requests again indicates very few police-recorded FGC/M-related crimes and almost no prosecutions, despite the government’s adoption of a strongly pro-prosecution approach. There were 28 alleged failures to protect a child from risk of FGC/M reported to the police between 2009 and 2018, and three alleged breaches of an FGM Public Protection Order (FGMPOs are designed to protect children who are considered at risk of FGC/M, including in cases where there may be an attempt to take them abroad for the procedure). None of these reports resulted in a charge or summons. Of the 173 alleged offences reported under the Female Genital Mutilation (FGM) Act 2003, only two proceeded to charge and neither were found guilty. Twenty-four alleged offences under the FGM Act 2003, four cases of alleged failure to protect a child from risk of FGC/M, and three alleged breaches of an FGMPO remained under investigation when these data were provided. Moreover, as databases do not record the date the alleged offence took place, we cannot confirm whether these reports concern incidents that are recent or historical.

Criminal case attrition, which refers to cases dropping out at any of the many exit points which exist within the criminal justice system, (see for example: Hester, M. and Lilley, S.J. (2017) ‘Rape Investigation and Attrition in Acquaintance, Domestic Violence and Historical Rape Cases: Attrition in Acquaintance, Domestic Violence and Historical Rape Cases’, Journal of Investigative Psychology and Offender Profiling, 14(2), pp. 175–188. doi: 10.1002/jip.1469) will invariably affect these figures. Whilst UK research on FGM case attrition is lacking, a recent exhaustive analysis of criminal investigations regarding suspected FGC/M in Sweden, which similarly has mandatory reporting, and based on 122 police reports, revealed that only three led to convictions [7]. Factors for case attrition included that: the level of suspicion was too high to warrant further action; conflicting statements by parents creating doubt in the evidence; evidence that FGC/M had taken place prior to migration to Sweden (and that therefore no crime had taken place); and that the medical examination of girls’ genitals had produced no evidence of FGC/M. Notwithstanding that the police reports may not have captured all FGC/M offences, still in light of the data and the fact that Swedish society is on high alert to the issue, the author of the study questioned whether coercive interventions (which include the detention of parents, compulsory medical examinations of girls, and the removal of girls from their homes into state care), are a proportionate response to the harm experienced by girls.

The Crown Prosecution Service reports one conviction from five prosecutions between 2010 and March 2019 [16, 17]. There is a clear discrepancy between the prosecution rates reported by the Crown Prosecution Service (*n* = 5) and Home Office (*n* = 2). The reason for this is unclear. It is possible that some of the Crown Prosecution Service reported cases were brought under legislation other than the 2003 FGM Act, on which the Home Office data focuses (https://www.cps.gov.uk/legal-guidance/female-genital-mutilation-prosecution-guidance). These figures may also be affected by limitations to the reporting systems on which these assessments are based. Police forces were not required to submit complete offence-level data to the Home Office prior to 2014. The Crown Prosecution Service also ‘does not collate formal statistics in relation to FGM’ [16]. Relevant information from the Ministry of Justice was also not held centrally nor electronically so was unavailable for this particular investigation.

The Ministry of Justice does provide data on FGM Public Protection Orders (FGMPO) and reveals that there have been 584 FGMPOs made between their introduction in July 2015 and the end of March 2020 [18]. There were no proceedings for any breaches of a FGMPO by March 2019. FGMPOs are civil orders issued by the family courts and they are designed to protect girls from the potential risk of experiencing FGC/M. They require a lower burden of proof, and in theory should be easier to enforce than criminal sanctions. Given the strong desire of governments to legislate and enforce the law in this area, there are however important questions to be asked about the low number of applications and disposals of FGMPOs [19]. But again, one plausible contributory factor is that the level of FGC/M risk to UK-based individuals is much smaller than suggested (even in relation to children being taken abroad for the procedure).

According to the Department for Education, FGC/M was identified as a risk factor at assessment in 1910 referrals to children’s social services between 1 April 2016 (when records began) and 31 March 2018 but there is no information regarding the evidential basis of these assessments or its reliability. Despite these data limitations, statistics from the Crown Prosecution Service, Home Office, Ministry of Justice, and Department for Education all suggest that the scale of the FGC/M risk existing in the UK is much smaller than has been suggested.

The National Health Services’ (NHS) ‘FGM Enhanced Dataset’ has collected data on FGC/M within the patient population from acute and mental health trusts and GP practices since 2015. (While the FGM Information Sharing System (FGM-IS, https://digital.nhs.uk/services/female-genital-mutilation-risk-indication-system-fgm-ris; https://assets.publishing.service.gov.uk/government/uploads/system/uploads/attachment_data/file/525390/FGM_safeguarding_report_A.pdf) flags the summary care record of any female infant born in England into a family with a history of FGC/M (on either parents’ side), the limitations of such approaches for establishing FGC/M risk have been discussed above). Submissions to the FGM Enhanced Dataset are required whenever an experience of FGC/M is suspected by a medical professional or on any patient receiving FGC/M-related treatment, including a change in FGC/M type, such as deinfibulation. The FGM Enhanced Database therefore cannot be used to directly estimate national prevalence as it only surveys the small subsection of the population who are seen by a medical professional. Early versions of data returns also collected information on the numbers of daughters born to women identified as having had FGC/M. This was intended to give some detail about the population potentially at risk, however, this approach since been revised. NHS Digital is currently working to remove these cases from the Enhanced Dataset but they remain in the figures presented here.

There are significant problems with data completeness. Until March 2020, only 2.5% of GP practices and 62.7% of NHS Trusts had ever submitted information to the Dataset [20]. The reasons for this are unclear as we cannot establish from the Dataset whether certain practices/trusts identified no cases, or there are cases which have gone unreported. A particular impediment for assessing prevalence is the decision not to record women who are asked but have not experienced FGC/M, or who have been recorded on the Dataset but subsequently died or migrated from the UK, which prevents the calculation of accurate figures regarding the population affected. Earlier approaches to data collection could count multiple attendances by the same individual, although there are now attempts to mitigate this using patients’ NHS numbers [21]. The preponderance of cases identified through maternity services would suggest some undercounting of women who do not become pregnant or receive maternity care when they do. Individuals are able to refuse submission of their information or subsequently request that their information is removed from the Dataset, which again undermines the value of the resource for assessing prevalence [21].

Submissions of required information are also often incomplete. Only 22% of women on the Dataset in 19/20 had complete data regarding their FGC/M type (see Box 1) and their age and location when it was undertaken [20]. There is evidence to suggest that reliance on self-reported FGC/M status may also introduce inaccuracies [22]. Finally, data suppression methods used to ensure anonymity by limiting our potential to identify the exact number of individuals affected limit their use for developing estimates of prevalence and risk particularly when numbers are small. Between 2015 and 2017, numbers of cases between 0 and 4 were presented in reports as ‘*’. Since then, numbers of cases between 1 and 7 are rounded to five, with all other numbers rounded to the nearest five. As such, a reported total of 15 could represent anywhere between 3 and 21 actual individuals.

Bearing in mind these caveats, between April 2015 and March 2020, 24,420 individual women and girls were identified through the Dataset as having had FGC/M at some point in their lives [20]. Notably, fewer than 5% of these incidents were reported as having taken place in the UK, the vast majority occurring before they migrated to the UK. This equates to ~425 women/girls, although data suppression techniques mean this is likely to be an over-estimate of the actual number of cases recorded. Of these, where the age is known, all those recorded between April 2015 and March 2017 (55/55), 82% of those recorded between April 2017 and March 2019 (70/85) and 92% of those recorded between April 2019 and March 2020 (110/120) were aged 18 or over at the time they had had FGC/M [20]. This may correspond to as few as eight girls who had FGC/M while resident in the UK and aged under 18 and recorded on the Dataset since these records began.

Moreover, where known, over 80% (795/920) of FGC/M cases among those born in, or having experiences of FGC/M, in the UK and recorded on the Database since April 2016 are type 4 (genital piercing, pricking, incising, cauterising or scraping for non-medical reasons) [20]. Moreover, around three-quarters (340/455) of incidents of FGC/M type 4 recorded on the Dataset involved genital piercing, which, when undertaken consensually by an adult woman is widely considered to be a form of cosmetic ‘enhancement’ rather than mutilation. As such, the number of women/girls recorded on the Dataset as experiencing FGC/M types 1, 2 or 3—often considered the more harmful forms—in the UK may be as few as one or two. Epidemiological surveillance studies of clinic populations which do not suffer from the same data limitations also indicate that the numbers of cases of FGC/M occurring in the UK among children are low, particularly type 3 which tends to dominate public and popular discussions of FGC/M [3, 23–26].

To summarise, currently available evidence—corroborated across multiple sources—indicates that the number of cases of FGC/M experienced by girls aged under the age of 18 and living in the UK is very low. While some, even many, cases are likely to be missing from these figures, this evidence would suggest that the risk to children living in the UK is well below the ‘tens of thousands’ reported by the Government and media. Current policy and practice relating to FGC/M in the UK may therefore be based on inaccurate evidence.

There is a need to protect children at risk from harm. However, our review of the available evidence points to clear limitations with the data and any policy on which it is based. Moreover, the assumption inherent to current approaches to FGM-safeguarding which assume high prevalence among certain communities appears unreliable. Such approaches can actively contribute to the stigmatisation, discrimination and criminalisation of individual children, their parents and families, and their communities [5, 6].

Research suggests that there has been a dramatic decline in the popularity of FGC/M among those with heritage in FGC/M-practising groups living in low prevalence countries, in part due to the success of national and community-level educational initiatives, many of them organised by people from FGC/M-affected groups themselves [1–6]. However, perceptions, such as those described above, that FGC/M remains valued and widespread among certain UK residents encourages responses to the protection of those perceived to be at risk which are at their best over-zealous. As a consequence, existing FGM-safeguarding policies are continuing to have a direct and significant negative impact on the lives of innocent families.

It appears unlikely that an accurate assessment of the prevalence and risk of FGC/M among the UK-resident population can be established using any of the methods examined here. While a range of data are collected related to those with experience, or at risk, of FGC/M, these lack value as a basis for population estimates as a consequence of data incompleteness, and also simply because they are not designed for this purpose.

We need to develop a bespoke mechanism which can better determine the attitudes, knowledge, and experiences of FGC/M of those living in the UK today, in partnership with those with heritage in FGC/M-affected communities. Only then can we more reliably understand practices of cutting and how they manifest in the context of changing social, political and legal dynamics.
